# Detection of *De Novo PAX2* Variants and Phenotypes in Chinese Population: A Single-Center Study

**DOI:** 10.3389/fgene.2022.799562

**Published:** 2022-03-31

**Authors:** Hua-Ying Xiong, Yong-Qi Shi, Cheng Zhong, Qin Yang, Gaofu Zhang, Haiping Yang, Daoqi Wu, Yaxi Chen, Qiu Li, Mo Wang

**Affiliations:** ^1^ Department of Nephrology, Children’s Hospital of Chongqing Medical University, Chongqing, China; ^2^ Chongqing Key Laboratory of Pediatrics, Ministry of Education Key Laboratory of Child Development and Disorders, China International Science and Technology Cooperation Base of Child Development and Critical Disorders, National Clinical Research Center for Child Health and Disorders, Pediatric Research Institute, Chongqing, China; ^3^ Key Laboratory of Molecular Biology for Infectious Diseases, Department of Infectious Diseases, Ministry of Education, Centre for Lipid Research, The Second Affiliated Hospital, Institute for Viral Hepatitis, Chongqing Medical University, Chongqing, China

**Keywords:** *PAX2* gene, novel variant, children, C1q nephropathy, kidney hypoplasia

## Abstract

**Background:** *PAX2* is a nuclear transcription factor gene that is highly conserved among species. Variants within *PAX2* could result in optic nerve colobomas and kidney hypoplasia. However, little clinical and genetic information is currently available about *PAX2* variants in Chinese children.

**Objective:** This study aims to further understand the clinical manifestations and genetic characteristics of *PAX2* variants in Chinese population.

**Methods:** In this single-center retrospective study, we analyzed the clinical data of 10 children identified as carriers of *PAX2* variants by gene sequencing. All the variants found in this study were analyzed using *in silico* prediction and American College of Medical Genetics and Genomics (ACMG) standards and guidelines.

**Results:** The mean age for developing the first symptom in 10 unrelated children was 7.2 years old. Proteinuria and bilateral kidney dysplasia were found in every patient. Two children underwent kidney histological examination; one child showed high-intensity C1q deposition in the kidney, and the other child showed focal segmental glomerular sclerosis (FSGS). Three children had *PAX2*-related ocular abnormalities, including nystagmus, retinal exudation, amblyopia, microphthalmia, microcornea, and total blindness. In addition, one patient had the comorbidity of oculocutaneous albinism (OCA). Eight different *PAX2* variants were found in ten patients, three of which were reported for the first time.

**Conclusion:** We reported some patients with unique manifestations and comorbidities, and we reported three variants that have not been previously identified. The *PAX2* gene is prone to spontaneous variants, and the outcome of patients is unfavorable. Because of the lack of specific therapy, genetic testing should be recommended for individuals with obvious evidence of kidney dysplasia and eye abnormalities, and kidney protective treatment should be initiated early.

## Introduction

*PAX2* is a member of the PAX family of transcription factors, which localizes to human chromosome band 10q24, spans 84.2 kb, and contains 12 coding exons (Rossanti et al., 2020). It is usually expressed in the urogenital system, eye, ear, and central nervous system. Animal model findings show that *PAX2* plays a vital role in cellular regeneration and organ development (Bower et al., 2012; Naiman et al., 2017; Zhang et al., 2018). *PAX2* variants are inherited in an autosomal dominant fashion, and these variants were initially characterized through the presence of kidney dysplasia and optic nerve abnormalities.

Patients with a homozygous variant of *PAX2* die soon after birth due to the lack of organogenesis; in contrast, patients with a heterozygous variant of *PAX2* may present with renal coloboma syndrome (RCS) and FSGS (Grimley and Dressler, 2018; Deng et al., 2019). Abnormal kidney structure or function (92% of individuals) and ophthalmological abnormalities (77% of individuals) have been reported (Zhang et al., 2018). Reported kidney findings with the *PAX2* variants include kidney hypoplasia, multicystic dysplastic kidney, horseshoe kidney, oligomeganephronia, and vesicoureteral reflux (VUR). Many patients eventually develop kidney failure (KF) and need kidney replacement therapy. However, visual impairment is known to have a wide variation that can be asymmetric, ranging from normal to complete blindness (Deng et al., 2019). In addition to kidney and ocular anomalies, some rare clinical features have been reported in patients with *PAX2* variants, such as hearing loss, skeletal deformities, growth retardation, microcephaly, heart defects, ovarian teratoma, and gout (Bower et al., 2012; Deng et al., 2019).

Although there have been several reports on *PAX2* variants in Chinese children, the reports are similar to other previous works in the literature. In this article, we will present the cases of 10 patients with *PAX2* variants with widely different clinical manifestations and some novel variants. Their kidney histological results are entirely different, and some patients have unique manifestations and comorbidities such as C1q kidney deposition and OCA. Our study further explores the clinical manifestations and genetic characteristics of *PAX2* variants in the Chinese population.

## Materials and Methods

### Subjects

We enrolled 10 patients with a *PAX2* (NM_003,990.4) variant by gene sequencing in our single-center study from May 2017 to April 2021. In our cohort, six patients were admitted to the hospital with proteinuria, three manifested abnormal kidney function, and one presented with kidney ultrasound abnormalities. Before the genetic tests, informed consent was obtained from all families. In addition to these ten patients, we also found one patient with recurrent painless gross hematuria, who had left renal vein entrapment (LRVE) and a *PAX2* variant (c.725G > A). However, his kidney structure and function were normal, and the pathogenicity of his variant was uncertain. His symptoms were totally relieved after conservative medical treatment. This result suggested that the LRVE, instead of the *PAX2* variant, was the leading cause of his gross hematuria. Because he did not have a confirmed genetic diagnosis, we finally excluded this patient.

### Clinical Assessment

We retrospectively reviewed the clinical findings of 10 patients, including the onset age, kidney findings, extrarenal manifestations, and kidney histological examination findings. The definitions of the main clinical manifestations, including kidney and ocular symptoms, and the methods of evaluating kidney function are shown in Supplementary Table S1 (Ketteler et al., 2018). The indications for gene sequencing were as follows: 1) highly suspected inherited metabolic diseases; 2) significant urinary system malformation; 3) nephropathy with poor response to corticosteroid therapy; and 4) kidney tubule disease. The indications for kidney histological examination were as follows: 1) recurrent proteinuria and/or glomerular hematuria without a definite cause; 2) worsened kidney function without a definite cause; 3) diagnosed with glomerulopathies, such as Henoch-Schonlein nephritis, IgA nephropathy, lupus nephritis, and anti-neutrophilic cytoplasmic antibody nephritis; and 4) nephropathy with poor response to corticosteroid therapy.

### Gene Sequencing and Genetic Analyses

Peripheral blood samples were collected from 10 patients and their parents, and genomic DNA was extracted according to standard procedures. The standard Illumina libraries were prepared by using a DNA Sample Prep Reagent Set (MyGenostics, Beijing, China). The amplified DNA was captured using the capture kit of urinary system genes. The enrichment libraries were sequenced on Illumina HiSeq 2000, Illumina Nova6000, and MGI DNBSEQ-T7. The raw data were saved in FASTQ format after sequencing. Both Illumina sequencing adapters and low-quality reads (<80 bp) were filtered by cut adaptor software. BWA was used to map the clean reads to the UCSC hg19 human reference genome.

All variants were identified by GATK or Sentieon and then annotated by ANNOVAR software, as well as the following multiple databases: 1,000 Genomes Project, ESP6500, ExAC, and ExAC-EAS. In downstream analysis, we used five steps to select the potential pathogenic mutations: 1) The mutation reads had to be more than 5, and the mutation ratio had to be no less than 30%. 2) The mutations for which the frequencies were more than 5% in the 1000g, ESP6500 ExAC, and ExAC-EAS database were removed. 3) If the mutations existed in InNormal database (MyGenostics), then they were dropped. 4) The synonymous mutations were removed. 5) After (1), (2), and (3), if the mutations were synonymous and they were reported in HGMD, then they were retained. Then, we performed pathogenicity analysis of the variants according to the ACMG standards and guidelines, and variants were classified as pathogenic, likely pathogenic, uncertain significance, likely benign, or benign (Richards et al., 2015). The ACMG guidelines and the matching degree between the patient’s phenotype and the gene-related disease were taken into consideration when identifying the pathogenic variant. *In silico* prediction of the variants was calculated from the output of the programs Sorting Intolerant from Tolerant (SIFT), Polymorphism Phenotyping v2 (PolyPhen_2), MutationTaster, GERP++, and REVEL. In addition, genomic DNA from all patients’ parents was obtained for Sanger sequencing, which was used to confirm the variants identified by next-generation sequencing (NGS).

## Results

### General Information

A total of 10 patients were enrolled in this study, including six boys and four girls, and their cases were sporadic. The ages of the patients on admission ranged from 0.3 to 15.2 years, with an average age of 8.8 years, while the age at the development of the first symptom was 7.2 (ranging from postnatal day to 14.7) years old. Six patients (patients 2, 3, 4, 5, 6, and 8) were identified with proteinuria as the first symptom, three patients (patients 1, 7, and 10) were admitted to the hospital due to KF, and kidney hypoplasia was detected in patient 9 after birth. Every patient in our cohort had confirmed congenital kidney hypoplasia and decreased estimated glomerular filtration rate (eGFR).

### Clinical Manifestations and Follow-Up

Proteinuria is the most common kidney manifestation in children with *PAX2* variants. Ten patients had varying degrees of proteinuria in our study; three of them (30.0%) presented nephrotic-range proteinuria. Six patients (60.0%) were found to have hematuria. In general, the patients with the *PAX2* variants had various clinical manifestations except for proteinuria, but the other nephrotic manifestations, such as edema and hypoproteinemia, were rare. Bilateral kidney dysplasia without ureterectasia was found in all patients by kidney ultrasound examination. Kidney cysts were detected in three patients (30.0%); one patient had multiple cysts, and the other two had a single cyst. The kidney ultrasound results of patients in our single-center study showed that the more severe the kidney dysplasia was, the worse kidney function of the patient. The relevant information is summarized in Table 1.

**TABLE 1:** Kidney phenotypes of patients with variants in *PAX2*.

Patient (No.)	1	2	3	4	5	6	7	8	9	10
Gender	F	M	M	F	M	F	M	F	M	M
Onset age (years)	13.0	9.7	8.0	3.0	6.3	0.2	14.7	9.7	Postnatal day	7.0
Kidney manifestations										
Chief complaint	Dermal ecchymosis and seizures	Proteinuria, hypertension	Proteinuria	Proteinuria	Proteinuria	Proteinuria	Pale, weak, oliguria, and edema	Proteinuria	Kidney ultrasound abnormality	Acroparesthesia, weak, gait abnormality
Age of abnormal Scr/onset of KF (years)	13.2	10.4	8.8	9.5	7.1	3.4	15.2	-	-	12.0
Hematuria	MHU	MHU	-	MHU	-	-	MHU	MHU	MHU	-
Proteinuria	+	NPU	+	NPU	+	+	+	+	+	NPU
24 h urinary protein (g/24 h)	1.2	4.6	1.0	2.8	0.3	0.7	NA	0.8	0.2	2.1
eGFR (ml/min/1.73 m2)	3.8	53.0	16.2	14.5	55.6	44.0	0.6	73.3	NA	3.4
Kidney ultrasound										
Age at kidney ultrasound (years)	13.0	10.0	9.0	9.5	7.0	3.4	15.0	9.0	0.3	12.4
Bilateral kidney hypoplasia	+	+	+	+	+	+	+	+	+	+
Kidney cysts	-	-	-	Single	-	Multiple	-	-	Single	-
Others	-	-	-	-	-	-	-	-	-	-
Kidney pathology										
Glomerulus	NA	NA	20% (1/5) glomerular segmental sclerosis located at the vascular pole and mesangial cells diffuse hyperplasia	NA	NA	NA	NA	No glomerular sclerosis, and mesangial cells hyperplasia	NA	NA
Kidney tubule	NA	NA	Granular and vacuolar degeneration of TEC and no atrophy	NA	NA	NA	NA	Granular degeneration and sloughing of TEC, loss of brush border, and partial tubules atrophy	NA	NA
Kidney interstitial	NA	NA	No obvious infiltration of inflammatory cells and fibrosis	NA	NA	NA	NA	No obvious infiltration of inflammatory cells and fibrosis	NA	NA
Immunofluorescence	NA	NA	-	NA	NA	NA	NA	High-intensity C1q deposition	NA	NA

F, female; M, male; PU, proteinuria; NPU, nephrotic-range proteinuria; GHU, gross hematuria; MHU, microscopic hematuria; eGFR, estimated glomerular filtration rate; TEC, tubular epithelial cell; NA, not available.

**FIGURE 1:**
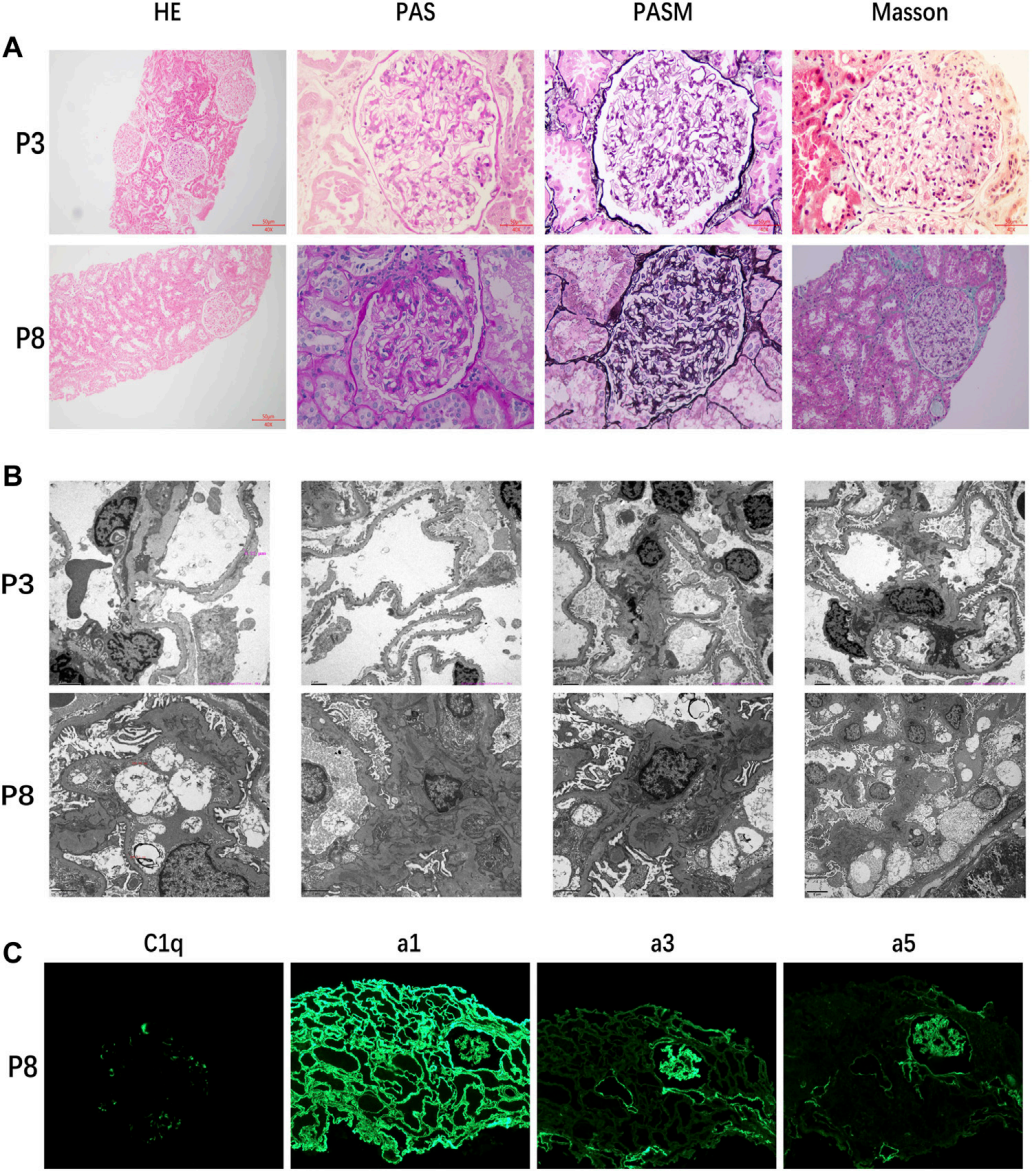
**(A)** Kidney histological examination of patient 3 showed FSGS and that of patient 8 showed mild proliferation of mesangial cells and stroma. **(B)** Electron microscopic results of patient 3 still supported FSGS and those of patient 8 showed high-density electron-dense deposition. **(C)** Results of immunofluorescence of patient 8 indicated diffuse high-intensity C1q deposition.

Only two patients underwent histological examinations, but the results were completely different (Figure 1). The kidney function of patient 3 deteriorated rapidly after onset, and histological examination indicated FSGS. In contrast, the kidney function of patient 8 was stable. The histological examination of patient 8 showed diffuse high-intensity C1q deposition in the mesangial area on immunofluorescence and a mild proliferation of mesangial cells and the stroma under light and electron microscopy.

Ocular manifestations are the most common extrarenal manifestations in children with *PAX2* variants. In our cohort, only patients 2 and 8 had subjective ocular abnormalities. Five patients (patients 1, 2, 3, 5, and 7) underwent ophthalmic fundus examination. Patient 2 presented with severe ocular lesions since birth, including microphthalmia, microcornea, congenital amblyopia of the right eye, and congenital blindness of the left eye. After hospitalization, bilateral conjunctival concretions were also discovered in patient 2. Retinal exudation without macular coloboma was detected in patient 7. Furthermore, patient 8 had binocular nystagmus, and the results of the brainstem auditory evoked potential (BAEP) were abnormal, showing suspicious bilateral distal auditory nerves or cochlear dysfunction. In addition to ocular and auditory lesions, malnutrition, obesity, short stature, growth retardation, cholecystolithiasis, and testicular dysgenesis could be observed in our group. Among them, cholecystolithiasis and testicular dysgenesis were rare extrarenal phenotypes that have not been reported before. The relevant information is summarized in Table 2.

**TABLE 2:** Extrarenal manifestations of patients with variants in *PAX2*.

Patient (No.)	1	2	3	4	5	6	7	8	9	10
Gender	F	M	M	F	M	F	M	F	M	M
Onset age (years)	13.0	9.7	8.0	3.0	6.3	0.2	14.7	9.7	Postnatal day	7.0
Ophthalmological findings										
Retinal abnormality	-	-	-	NA	-	NA	Retinal exudation	NA	NA	NA
Microphthalmia	-	Left	-	-	-	-	-	-	-	-
Microcornea	-	Left	-	-	-	-	-	-	-	-
Blindness	-	Left	-	-	-	-	-	-	-	-
Amblyopia	-	Right	-	-	-	-	-	-	-	-
Nystagmus	-	-	-	-	-	-	-	Bilateral	-	-
Conjunctival concretion	-	Bilateral	-	-	-	-	-	-	-	-
Others										
Other findings	-	Obesity, fair skin, light yellow hair, and eyebrows	-	Obesity and short stature	Growth retardation and short stature	-	-	Cholecystolithiasis	Testicular dysgenesis	-
BAEP	-	-	NA	NA	-	NA	NA	Suspicious bilateral distal auditory nerves or cochlea dysfunction	NA	NA

Note: F, female; M, male; BAEP, brainstem auditory evoked potential; NA, not available.

It is worth mentioning that we revealed suspicious OCA in patient 2, such as fair skin, light yellow hair, and eyebrows. The gene test of this patient revealed compound heterozygous variants in the OCA2 gene (c.1441G>A, c.727C>T), originating from his father and his mother, respectively. The variants have both been reported as pathological variants of OCA (Lee et al., 1994; Wei et al., 2010; Hwang et al., 2014; Bullich et al., 2018; Ishiwa et al., 2019). This meant that patient 2 could also be diagnosed with OCA, except the *PAX2* variant. The ocular symptoms of OCA are mainly reduced pigmentation and congenital nystagmus, which were inconspicuous in patient 2. Therefore, we supposed that the *PAX2* variant was the leading cause of the ocular lesions of patient 2.

In our single-center study, the longest follow-up time of 10 children was 4.4 years, the shortest was 0.67 years, and the average was 2.3 years. Among them, five patients (patients 1, 3, 4, 7, and 10) had developed KF, and the kidney function of three patients deteriorated drastically within a few months but that of the other two patients deteriorated over several years. However, almost all five patients were in adolescence at the onset of KF, and their onset ages ranged from 8.8 to 15.2 years. The kidney function of the remaining five patients was relatively stable. To date, four patients (patients 1, 4, 7, and 10) are undergoing maintenance hemodialysis. The other six patients are receiving oral drug treatment to protect kidney function. None of the patients had new ocular abnormalities.

### Pathogenic Variants in the *PAX2* Gene

**FIGURE 2:**
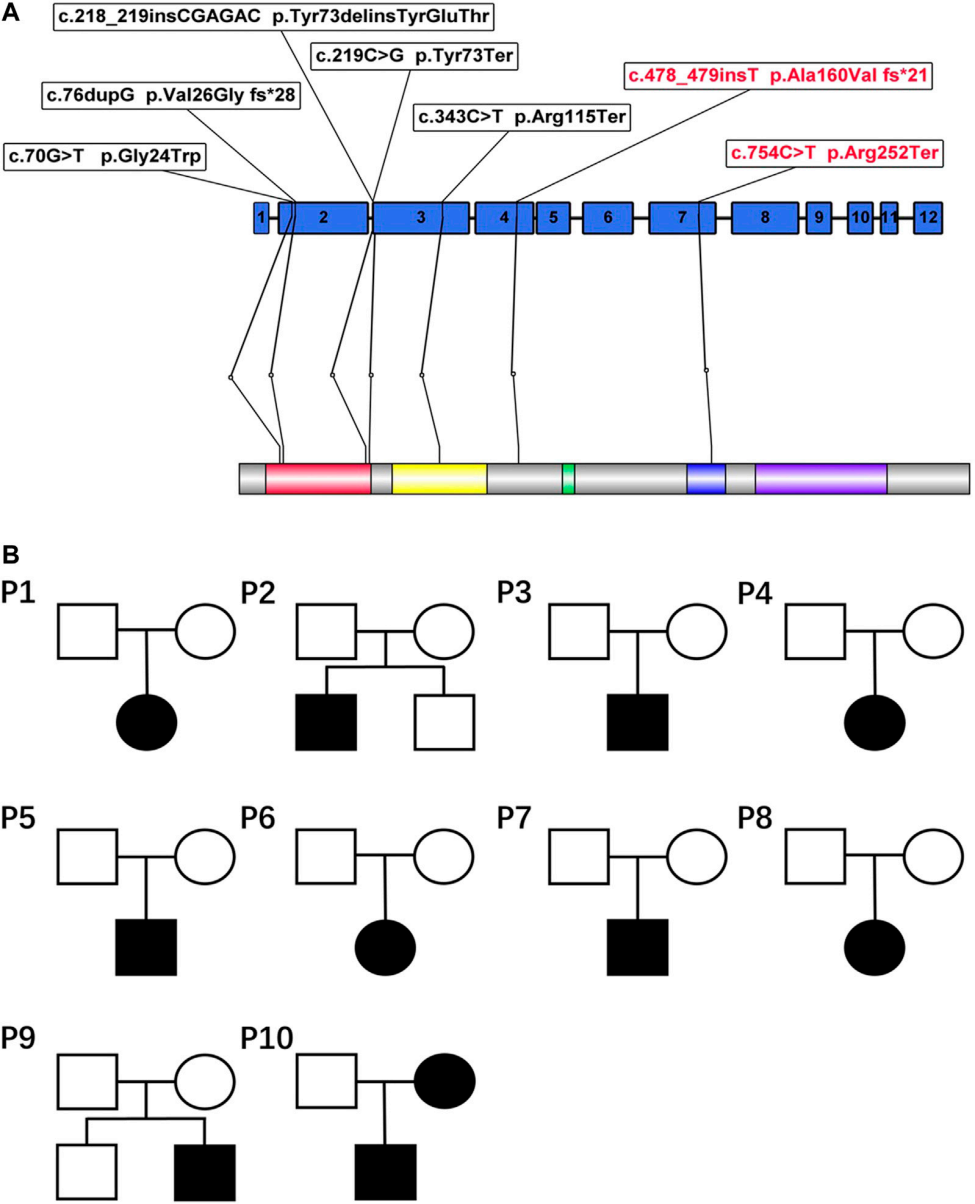
**(A)** PAX2 domain structure and localization of seven variants in this article. The variants marked with red in the figure refer to the novel variants reported for the first time. PAX2 is characterized by an N-terminal paired domain consisting of the N terminus (red) and C terminus (yellow). The relative locations of the other domains are also indicated, including the octapeptide motif (green), the homeodomain(blue), and a transactivation domain (violet). **(B)** Pedigrees of 10 families. The genetic variants of patient 10 originated from the mother, and his mother had clinical manifestations. The variant of patient 4 originated from her father, whose manifestations were not obvious.

Eight different variants, most of which occurred in exons 2–4, were found in the 10 patients. Among all the variants, variants c.754C > T and c.478_479insT and the large genomic deletions of patient 4 were not previously reported in the Human Gene Variant Database (HGMD) or ClinVar (Figure 2). According to ACMG standards, two novel variants (c.754C>T and c.478_479insT) were identified as pathogenic variants, and the remaining variant (genomic deletions) was considered to be likely pathogenic. The details and bioinformatic analyses of all the *PAX2* variants are summarized in Table 3. We validated two novel variants, and the secondary protein structure of those variants was changed, which is shown in Supplementary Figure S1.

**TABLE 3:** Genetic characteristic and bioinformatic analyses of eight *PAX2* variants.

Nucleotide change	Deduced protein change	Location	Zygosity (segregation)	ACMG standards	SIFT		Polyphen2		MutationTaster		REVEL		GERP++	Patient (No.)
					Prediction	Score	Prediction	Score	Prediction	Score	Prediction	Score	Prediction	
c.76dupG	p. Val26Gly f s*28	Exon 2	Het (N)	Pathogenic: PVS1+PS2+PS4+PM2	_	_	_	_	Disease causing	1.00	_	_	_	1, 2 and 9
c.754C > T	p. Arg252Ter	Exon 7	Het (N)	Pathogenic: PVS1+PS2+PM2	_	_	_	_	Disease causing	1.00	_	_	Nonconserved	3
c.343C > T	p. Arg115Ter	Exon 3	Het (N)	Pathogenic: PVS1+PS1+PS2+PM2	_	_	_	_	Disease causing	1.00	_	_	Conserved	5
c.70G > T	p. Gly24Trp	Exon 2	Het (N)	Pathogenic: PS1+PS2+PM1+PM2+PP3	Damaging	0.00	Probably damaging	1.00	Disease causing	0.99	Deleterious	0.91	Conserved	6
c.478_479insT	p. Ala160Val fs*21	Exon 4	Het (N)	Pathogenic: PVS1+PS2+PM2	_	_	_	_	Disease causing	1.00	_	_	_	7
c.218_219insCGAGAC	p. Tyr73delins	Exon 3	Het (NA)	Likely pathogenic: PS1+PM2+PM4	_	_	_	_	Disease causing	3.8e^−8^	_	_	_	8
	TyrGluThr													
c.219C > G	p. Tyr73Ter	Exon 3	Het (M)	Pathogenic: PVS1+PS4+PM2	_	_	_	_	Disease causing	1.00	_	_	Conserved	10
Genomic deletions	Gene deletion	Chr10(q24.31-q24.32)	Het (P)	Likely pathogenic	_	_	_	_	_	_	_	_	_	4

M, maternal; P, paternal; N, novel mutation; mutations could not be found in father or mother; NA, not available; ACMG, American College of Medical Genetics.

All the variants were heterozygous. Segregation analysis was performed in every patient and almost all first-degree relatives using Sanger sequencing. Most variants could not be detected in their first-degree relatives, suggesting that they may be *de novo* variants. The variants of two patients (patients 4 and 10) were confirmed to originate from their parents (Figure 2). The variant of patient 10 was derived from his mother. Although she had no obvious clinical symptoms, she also had proteinuria and decreased kidney function. The variant of patient 4 originated from her father, whose routine urine test results were normal.

## Discussion

This study presents ten patients with eight different *PAX2* variants, and three of these variants were reported for the first time. Every patient in our cohort had confirmed congenital kidney hypoplasia and decreased eGFR, and they all had proteinuria. One patient had a unique histological presentation of high-intensity C1q deposition, and one patient was found to have the comorbidity of OCA, which had not been previously reported. Furthermore, we also reported several novel manifestations of *PAX2* variants, including cholecystolithiasis and testicular dysgenesis.

Previous studies have reported that kidney hypoplasia and ocular abnormalities are the most common manifestations of *PAX2*-associated disease. Except for these symptoms, there may be rare manifestations such as intellectual disability, joint laxity, and sensorineural deafness (Bower et al., 2012; Liu et al., 2018). In our single-center study, kidney hypoplasia was detected in every patient, and several patients had ocular lesions. Among the ten patients, three patients (patients 1, 2, and 9) carried the same *PAX2* variant (c.76dupG), and they all had decreased eGFR and kidney hypoplasia. Nevertheless, the results of ophthalmic examinations were not the same. They were not related to each other, neither were they relatives. The variant (c.76dupG) was a recurring mutation that had been reported by many studies, rather than a common mutation derived from a single founder. Compared with the three patients in our study, some patients have been reported to have other presentations, such as microcephaly, mental retardation, soft skin, and VUR (Schimmenti et al., 1997; Ford et al., 2001). This phenomenon also agrees with our earlier observations, which showed that each individual might have a different phenotype even if they carry the same variant within the same family (Iatropoulos et al., 2012). Previous studies found that the *PAX2* gene is likely modulated by other genes, amplifying or reducing the genetic effect, which may be explained by haploinsufficiency (Barua et al., 2014). Although many studies have focused on the expression patterns of the mutated gene, several questions remain to be answered, such as incomplete penetrance and parental germline mosaicism. Our study’s *de novo* rate was 70.0%, higher than that reported (50%) previously (Bower et al., 2012). The small sample size may be the major reason.

Unexpectedly, we found that patient 8 with the *PAX2* variant was diagnosed with C1q nephropathy by kidney histological examination, while the gene test showed normal sequences of C1q-related genes. C1q nephropathy is rare, with prevalence ranging from 2.1 to 9.2% in pediatric biopsies (Devasahayam et al., 20152015). As far as we know, this is the first report of C1q nephropathy in a patient with a *PAX2* variant. However, the relationship between *PAX2* variants and C1q nephropathy is still unclear. It is recognized that *PAX2* variants are one of the important causes of FSGS, as reported by many studies (Rachwani Anil et al., 2019; Vivante et al., 2019; Nagano et al., 2020). The systemic activation of complement in primary FSGS has been identified (Huang et al., 2020). In a large study of C1qN, C1q deposition disappeared in some patients throughout the follow-up period, with FSGS developing in repeat biopsy samples (Hisano et al., 2008). As a transcription factor, *PAX2* has not been reported to be involved in the pathogenesis of immune diseases. Therefore, we suppose that more studies are needed to clarify the correlation between *PAX2* and C1q nephropathy, which could be a research direction in the future.

In our research, five patients (50.0%) developed KF, while the kidney function of the other patients was relatively stable. Although *PAX2* variants have been reported every year, it is still unclear what factors affect patients’ disease progression. Unlike most common kidney diseases, the onset age and the duration of progression to KF of this disease vary greatly. In some patients, kidney function deteriorates drastically within a few months, whereas in some patients, kidney function remains stable for decades (Rasmussen et al., 2018; Connaughton et al., 2019; Saida et al., 2020). Compared with the patients with other hereditary kidney diseases, patients with *PAX2* variants develop KF mainly in adolescence rather than in infancy or childhood.

There are some limitations to our study. First, our study was retrospective, and most of them had no subjective visual or hearing abnormalities. Therefore, not all the patients had undergone ocular and hearing examinations. Second, only two patients had received a kidney histological examination, and one of them was diagnosed with C1q nephropathy, which is most likely the first report to date. However, the reason for C1q deposition is still unknown and needs to be further explored. Third, except for immediate family members, the other family members’ information was not available in all the patients of our center. However, no suspicious family history was found.

In summary, our study identified three unrecognized variants, two patients with novel presentations, and several novel manifestations of the *PAX2* variants. Patients with *PAX2* variants developed KF mainly in adolescence rather than in early life, and the clinical manifestations of those patients were highly heterogeneous. Many patients with *PAX2* variants developed KF requiring long-term kidney replacement therapy and even transplantation. Thus, individuals with clear evidence of kidney hypoplasia and ocular abnormalities should be suggested for genetic testing. If such individuals have *PAX2* variants, they should be examined for PAX2-related organ damage, closely followed up for kidney function, professionally managed to avoid KF development, and indicated for appropriate genetic counseling.

## Data Availability

The datasets presented in this study can be found in online repositories. The names of the repository/repositories and accession number(s) can be found below: https://www.ncbi.nlm.nih.gov/, PRJNA773192.
