# Supplementary material for: Detection of De Novo PAX2 Variants and Phenotypes in Chinese Population: A Single-Center Study
Source: Front Genet. 2022 Mar 31;13:799562. doi: 10.3389/fgene.2022.799562 (PMC9014304; doi:10.3389/fgene.2022.799562)
Supplement: Supplementary file 1 [file DataSheet1.docx]

**Supplementary table 1** Definitions of clinical manifestations and important examinations

| Item | definition |
| --- | --- |
| Microscopic hematuria (MHU) | >3 red blood cells per high-power field on one urine sample under microscope, with no significant change in appearance |
| Gross hematuria (GHU) | the blood content per liter of urine reached or exceeded 1ml, with observable change in appearance |
| Proteinuria | proteinuria between 0.15g/day and 50mg/kg/day |
| Nephrotic proteinuria (NPU) | proteinuria ≥50mg/kg/day |
| Short stature | the height is lower than the 3th percentile of the height of children of the same sex, age |
| Obesity | the weight is greater than the 97th percentile of the weight of children of the same height |
| Hypertension | the systolic blood pressure and (or) diastolic blood pressure is greater than or equal to the 95th percentile of the blood pressure of children of the same sex, age, and height. |
| Oliguria | daily urination volume of school-age children is <400mL/m2, preschool children is <300mL/m2 |
| Renal hypoplasia | ultrasound detected lengths less than the mean for the corresponding age minus two standard deviations |
| left renal vein entrapment (LRVE) | the peak velocity (PV) ratio between the aortomesenteric portion (AMP) and renal hilum of the LRV was calculated, and a renal hilum PV greater than 5-fold the AMP |
| Chronic kidney disease (CKD) stages | divided into five stages according to eGFR: stage 1, >90 ml/ (min·1.73 m^2^); stage 2, 60–89 ml/ (min·1.73 m^2^); stage 3, 30–59 ml/ (min·1.73 m^2^); stage 4, 15–29 ml/ (min·1.73 m^2^); and stage 5, <15 ml/ (min·1.73 m^2^)^7^ |


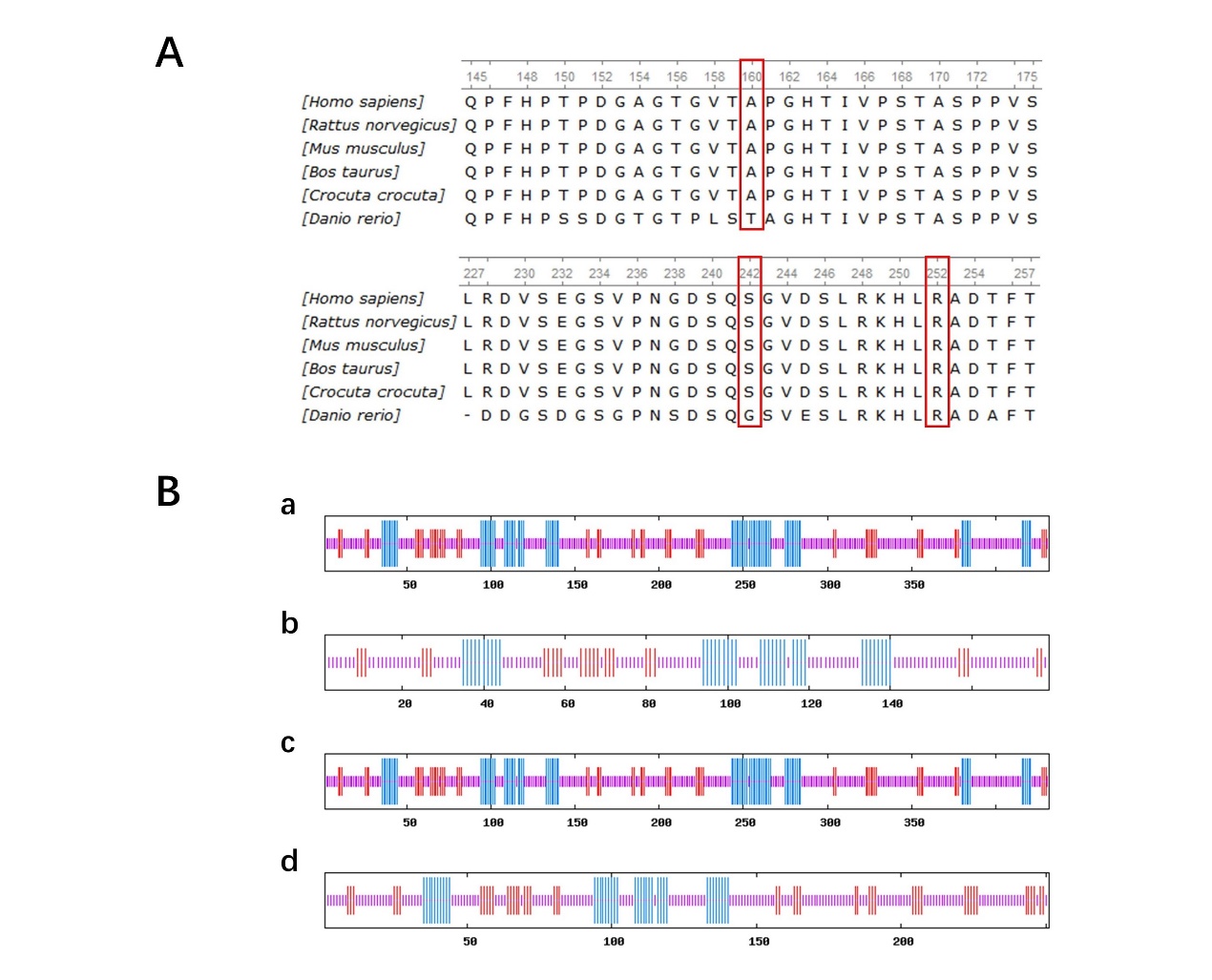


**Supplementary Figure 1** (A) The three variants (c.725G>A, c.754C>T, c.478_479insT), as marked with red in (fig. 2.) are localized in a highly conserved amino acid sequence among representative species. (B) In the wild type, the protein is composed of three secondary structures: alpha-helix (19.21%), extended strand (13.89%), and random coil (66.90%) (a). When the 160th position occurred the frameshift mutation, the second structure changed: alpha helix (21.23%), extended strand (15.08%), and random coil (63.69%) (b). When the 242nd position Ser mutated to Asn, the second structure changed: alpha helix (19.21%), extended strand (13.89%), and random coil (66.90%) (c). When the 252nd position Arg mutated, the second structure changed: alpha helix (15.14%), extended strand (18.73%), and random coil (66.14%) (d).
